# Endoplasmic reticulum stress in adipose tissue augments lipolysis

**DOI:** 10.1111/jcmm.12384

**Published:** 2014-11-08

**Authors:** Elena Bogdanovic, Nicole Kraus, David Patsouris, Li Diao, Vivian Wang, Abdikarim Abdullahi, Marc G Jeschke

**Affiliations:** Division of Plastic Surgery, Division of General Surgery, Department of Surgery, Department of Immunology, University of Toronto, Ross Tilley Burn Centre, Sunnybrook Health Sciences Centre, Sunnybrook Research InstituteToronto, ON, Canada

**Keywords:** ER stress, adipose tissue, lipolysis, fatty infiltration, hepatomegaly

## Abstract

The endoplasmic reticulum (ER) is an organelle important for protein synthesis and folding, lipid synthesis and Ca^2+^ homoeostasis. Consequently, ER stress or dysfunction affects numerous cellular processes and has been implicated as a contributing factor in several pathophysiological conditions. Tunicamycin induces ER stress in various cell types *in vitro* as well as *in vivo*. In mice, a hallmark of tunicamycin administration is the development of fatty livers within 24–48 hrs accompanied by hepatic ER stress. We hypothesized that tunicamycin would induce ER stress in adipose tissue that would lead to increased lipolysis and subsequently to fatty infiltration of the liver and hepatomegaly. Our results show that intraperitoneal administration of tunicamycin rapidly induced an ER stress response in adipose tissue that correlated with increased circulating free fatty acids (FFAs) and glycerol along with decreased adipose tissue mass and lipid droplet size. Furthermore, we found that in addition to fatty infiltration of the liver as well as hepatomegaly, lipid accumulation was also present in the heart, skeletal muscle and kidney. To corroborate our findings to a clinical setting, we examined adipose tissue from burned patients where increases in lipolysis and the development of fatty livers have been well documented. We found that burned patients displayed significant ER stress within adipose tissue and that ER stress augments lipolysis in cultured human adipocytes. Our results indicate a possible role for ER stress induced lipolysis in adipose tissue as an underlying mechanism contributing to increases in circulating FFAs and fatty infiltration into other organs.

## Introduction

The endoplasmic reticulum (ER) is a cell organelle that comprises the nuclear envelope and forms a continuous membranous network throughout the cytoplasm of cells [reviewed in [1]]. The ER is the site of protein synthesis and folding, lipid synthesis, drug detoxification, Ca^2+^ homoeostasis and signalling, and forms close contacts with other organelles such as the mitochondria, peroxisomes, lysosomes and lipid droplets [reviewed in [1–4]]. ER stress occurs when the functioning of the ER is impaired and unfolded or misfolded proteins accumulate within the lumen [5]. To restore homoeostasis and to counteract the protein burden, the ER initiates the unfolded protein response (UPR) *via* the activation of three transmembrane receptors in the ER membrane: activating transcription factor 6 (ATF6), inositol requiring enzyme 1α (IRE1α) and PRKR-like endoplasmic reticulum kinase (PERK) [6–8]. ATF6 increases the transcription of X-box-binding protein 1 (Xbp1) mRNA which is then cleaved by the endoribonuclease activity of IRE1α to generate the spliced form Xbp1s, a transcriptional activator of genes involved in the UPR [9,10]. In response to ER stress, activated PERK induces the expression of C/EBP-homologous protein (CHOP), a transcription factor with proapoptotic activity [11]. In resting cells, IRE1α, PERK and ATF6 are bound to the ER chaperone protein GRP78/BiP (78 kD glucose regulated protein/immunoglobulin heavy chain-binding protein homologue) on the luminal side [12,13]. The accumulation of unfolded or misfolded proteins in the lumen of the ER increases the expression of GRP78 and induces the dissociation of GRP78 from IRE1α, PERK and ATF6 leading to activation of the receptors and induction of the UPR [12–14]. Given the central role of the ER in cellular functioning and the numerous contacts the ER makes with other organelles, ER stress or dysfunction has been implicated as a mediating factor in several pathological conditions. For example, we have recently shown that severe illness such as a thermal injury induces ER stress in various tissues such as the liver and is accompanied by metabolic alterations such as hyperglycaemia, increased lipolysis and hepatomegaly [15–18]. Within the liver, ER stress leads to hepatocyte dysfunction, insulin resistance and apoptosis [15,19]. Our observation that a severe burn causes hepatic steatosis prompted us to examine the effects of ER stress in adipocytes and whether a burn induces ER stress in adipose tissue. To answer these questions we first induced ER stress *in vivo* and determined whether ER stress within adipose tissue contributes to hepatomegaly.

Numerous pharmacological agents interfere with the normal functioning of the ER and consequently induce ER dysfunction and ER stress. Tunicamycin, an antibiotic isolated from Streptomyces sp. that inhibits *N*-linked glycosylation of proteins and lipids within the ER [20,21], has been widely used to induce ER stress *in vivo* and *in vitro*. Intraperitoneal administration of tunicamycin in mice induces a robust ER stress response within the liver and kidney [5,22,23]. Most interestingly, mice injected with a single dose of tunicamycin develop profound fatty livers within 24–48 hrs with some lipid accumulation in the kidneys indicating that this process is somewhat specific to the liver [22–24]. We hypothesized that adipose tissue most likely plays an important role in this pathophysiological process. It has been shown that in cultured adipocytes ER stress stimulates lipolysis *in vitro via* activation of protein kinase A (PKA) and hormone sensitive lipase (HSL) [25,26]. We hypothesized that tunicamycin induces ER stress in adipose tissue that leads to increased lipolysis and subsequently to fatty infiltration of the liver. Therefore, the aim of our study was to determine whether tunicamycin administration in mice induces ER stress in adipose tissue and whether the rapid development of fatty livers following tunicamycin administration is due to increases in circulating free fatty acids (FFAs) arising from ER stress induced lipolysis. Determining the physiological mechanisms contributing to the development of fatty livers and hepatomegaly are clinically relevant since fatty infiltration of the liver and hepatomegaly are detrimental processes associated with poor outcomes in several human pathologies, particularly in burned patients [18,27].

## Materials and methods

### Induction of ER stress by tunicamycin

Male Balb/c mice (Taconics) were housed and cared for in accordance with the Guide for the Care and Use of Laboratory Animals. All procedures performed in this study were approved by the Sunnybrook Research Institute Animal Care Committee (Toronto, Ontario, Canada). Tunicamycin from Streptomyces sp. (Sigma-Aldrich, Oakville, ON, Canada) was dissolved in dimethyl sulfoxide (DMSO) and diluted in sterile 150 mM dextrose to obtain a tunicamycin concentration of 10 μg/μl. Male Balb/c mice (20–25 g) were injected intraperitoneally with tunicamycin solution (1 μg/g body mass) as described previously [5]. As controls, mice were injected intraperitoneally with control buffer (150 mM dextrose containing 1% DMSO).

### Isolation of primary hepatocytes

Primary mouse hepatocytes were isolated as described previously [28]. Briefly, hepatocytes were isolated from Balb/c mice by first perfusing the liver with Hank's balanced salt solution (HBSS; without Ca^2+^ or Mg^2+^) containing 500 μM EGTA and penicillin-streptomycin (pH 7.4) followed by perfusion with HBSS (containing Ca^2+^ and Mg^2+^), collagenase type 1 (0.3 mg/ml) and 2.5 mM HEPES (pH 7.4). The livers were excised, placed in cold DMEM (containing 1 g/l glucose, 10% FBS, penicillin-streptomycin) followed by filtration through a 100 μm cell strainer. The cells were washed twice and cultured in the same medium on collagen coated tissue culture dishes in a 37°C humidified incubator (95% air, 5% CO_2_).

### Immunoblotting

Tissues were homogenized in RIPA lysis buffer (50 mM Tris-HCl pH 7.5, 150 mM NaCl, 1% Igepal, 0.5% sodium deoxycholate, 0.1% SDS, 1 mM NaF and protease inhibitors) incubated on ice for 20 min. and centrifuged at 10,000 × g for 10 min. at 4°C. The infranatant was removed and proteins quantified using the BCA Protein Assay Kit (Pierce, Mississauga, ON, Canada). Proteins were resolved by SDS-PAGE followed by Western blotting using antibodies recognizing GRP78, IRE1α, eukaryotic translation initiation factor 2 subunit α (eIF2α), alpha/beta tubulin or glyceraldehyde-3-phosphate dehydrogenase (GAPDH). All antibodies were purchased from Cell Signaling (Whitby, ON, Canada). Species appropriate secondary antibodies conjugated to horse radish peroxidise (Bio-Rad, Mississauga, ON, Canada)) were used and proteins visualized by enhanced chemiluminescence using the Bio-Rad ChemiDoc MP Imaging System.

### Real-time quantitative PCR

Total RNA was extracted from epididymal fat pads using TRIzol-chloroform (Life Technologies, Burlington, ON, Canada) with subsequent purification using the RNeasy Kit (Qiagen, Toronto, ON, Canada) according to the manufacturer's instructions. RNA (2 μg) was transcribed to cDNA using the high capacity cDNA reverse transcription kit (Applied Biosystems, Burlington, ON, Canada). Real-time quantitative PCR was performed with the Applied Biosystems StepOnePlus Real-Time PCR System. The sequences of all primers are listed in Table S1.

### Oil red O staining

Tissues were perfused with phosphate-buffered saline, coated with OCT (optimal cutting temperature compound) (Tissue-Tek), placed on dry ice and stored at −80°C until further analysis. Frozen tissue blocks were sectioned 10 μm thick, mounted on slides and fixed in formaldehyde (40%) for 1 min. The slides were stained with Oil Red O for 10 min. at room temperature, rinsed with water and stained using Gill's haematoxylin for 1 min. The slides were washed with water, mounted with aqueous mounting medium and imaged using the Leica DM 2000 LED microscope. To stain primary hepatocytes, cells were washed with PBS and incubated in 10% formalin for 30 min. at room temperature. The cells were washed with deionized water and incubated in 60% isopropanol for 5 min. and subsequently stained with Oil Red O solution for 5 min. at room temperature. The cells were rinsed with tap water, counterstained using Gill's haematoxylin for 1 min. and imaged using the Zeiss Primo Vert inverted microscope.

### Transmission electron microscopy

Epididymal fat pads and liver tissue (3 mm pieces) were fixed in phosphate buffer containing 4% paraformaldehyde and 1% glutaraldehyde followed by post-fixation in phosphate buffer containing 1% osmium tetroxide. The tissues were dehydrated using a graded series of ethanol, washed with propylene oxide and infiltrated with Epon Araldite (E/A) resin using a graded series of E/A resin and propylene oxide. Following polymerization, tissue sections (70 μm thick) were counterstained using saturated uranyl acetate followed by lead citrate and imaged using the Hitachi (Japan) H7000 transmission electron microscope.

### Determination of free glycerol, FFA and triglyceride levels

Blood was collected from mice by cardiac puncture, transferred to tubes containing 0.5 M EDTA on ice, centrifuged at 1000 × g for 15 min. at 4°C and the plasma collected and stored at −80°C until further analysis. For the determination of free glycerol, plasma samples were diluted 10× and deproteinized using a 10 kD spin column (Abcam, Toronto, ON, Canada). Deproteinized plasma was further diluted 1:10,000 prior to analysis. Free glycerol concentrations in the plasma and cell culture media were determined using the Free Glycerol Detection Kit (Abcam) according to the manufacturer's instructions.

To determine the FFA concentrations in plasma and tissues, lipids were extracted using chloroform containing 1% Triton-X 100. The samples were centrifuged (1000 × g) for 10 min. and the lower lipid phase removed. The lipids were dried and quantified using the Free Fatty Acid Quantification Kit (BioVision, Burlington, ON, Canada) according to the manufacturer's instructions. For the quantification of FFAs in the heart, the lipid extracts were diluted in 300 μl assay buffer prior to analysis. For the quantification of FFAs in blood, lipids were extracted from 35 μl of plasma. For analysis of liver FFAs, lipids were extracted from 15 mg of liver tissue and re-suspended in 200 μl assay buffer, 25 μl was used for FFA determination. Triglyceride levels were determined using a commercially available kit (Abcam) according to manufacturer's instructions.

### Human adipose tissue samples and isolation of primary human adipocytes

Patients admitted to the Ross Tilley Burn Centre at Sunnybrook Hospital (Toronto, Canada) or patients undergoing elective surgery at Sunnybrook Hospital were consented pre-operatively for tissue collection. Approval for our study was obtained from the Research Ethics Board at Sunnybrook Hospital.

Subcutaneous white adipose tissue obtained from surgery was dissected to remove skin and muscle and either stored at −80°C for protein and RNA analysis or processed immediately to obtain isolated adipocytes.

Adipose tissue was suspended in RPMI-1640 media (Wisent Inc., Burlington, ON, Canada) containing 2 mg/ml collagenase type 1 (BioShop, Burlington, ON, Canada), minced with scissors and incubated in a 37°C water bath. Collagenase digested tissue was filtered through gauze and then through a 100 μm cell strainer followed by brief centrifugation to allow adipocytes to float to the top which were then collected. Cells were washed three times with RPMI media to remove collagenase, re-suspended in RPMI media and cultured in a 37°C humidified incubator (95% air, 5% CO_2_).

### Statistics

The Student's *t*-test was used to compare control and tunicamycin treated groups. Statistical significance was determined when *P* ≤ 0.05.

## Results

### Tunicamycin induces ER stress in adipose tissue

**Fig. 1:**
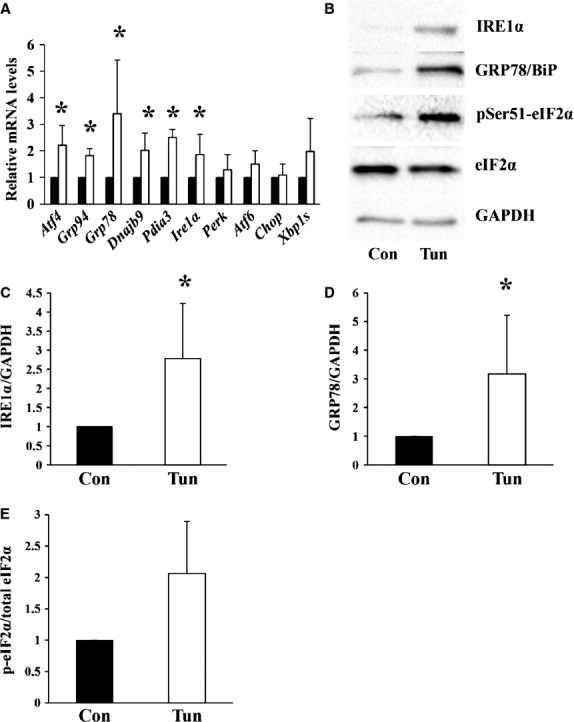
Male Balb/c mice were injected with either control buffer (Con) or tunicamycin (Tun). After 24 hrs, the epididymal fat pads were dissected and (**A**) total RNA was extracted and transcribed to cDNA. Relative mRNA levels of the indicated genes were quantified using real-time PCR and normalized to 18S rRNA. The value obtained for the control for each gene was assigned a value of 1. The value obtained following injection with tunicamycin was expressed relative to the control value. Results represent the mean ± SD of *n* = 3–8 experiments. ▪ Control, □ Tunicamycin. (**B**) Equal amounts of protein were resolved by SDS-PAGE followed by immunoblotting using antibodies recognizing the indicated proteins. For IRE1α, GRP78 and GAPDH intervening lanes have been removed. (**C**–**E**) The intensities of the bands in (**B**) were quantified from *n* = 3–6 experiments. The control values were set to 1. The values obtained from tunicamycin treated mice were expressed relative to the control. * Indicates *P* < 0.05.

The aim of the first part of this study was to examine whether tunicamycin induces ER stress in adipose tissue. Mice were injected with either control buffer or tunicamycin. After 24 hrs, the presence of ER stress was evaluated in the epididymal fat pads which served as a source of adipose tissue. We first examined a panel of ER stress markers by real-time quantitative PCR (Fig. 1A). Following tunicamycin injection, all ER stress markers examined were up-regulated although to varying extents (Fig. 1A). Of note was the high variability in the ER stress response in the epididymal fat pads 24 hrs following tunicamycin administration. There was significant up-regulation of *Atf4*, 94 kD glucose regulated protein (*Grp94*), *Grp78*, *Dnajb9* (DnaJ homologue subfamily B member 9), *Pdia3* (protein disulphide-isomerase A3) and *Ire1*α (Fig. 1A). We also examined ER stress in adipose tissue at the protein level by Western blotting by evaluating a subset of ER stress markers. There was significant up-regulation of IRE1α and GRP78 in adipose tissue following tunicamycin injection (Fig. 1 B–D). In the epididymal fat pads, increased levels IRE1α and GRP78 can be detected within 3 hrs after injection with tunicamycin (Fig. S1 and data not shown). Interestingly, although CHOP was up-regulated in the liver and kidney following tunicamycin administration, no detectable levels of CHOP were seen in adipose tissue (Fig. S2). We also examined the levels of PERK, the upstream activator of CHOP and found that while PERK was readily detectable in liver homogenates, the PERK protein was not detected in adipose tissue (Fig. S3). Although the PERK protein could not be detected in adipose tissue homogenates, possibly because of low levels of expression of the protein, signalling though the PERK pathway likely occurs demonstrated by the up-regulation of *Atf4* mRNA and increased phosphorylation of eIF2α on serine 51 (Fig. 1A, B and E).

### ER stress in adipose tissue is associated with increased lipolysis

**Fig. 2:**
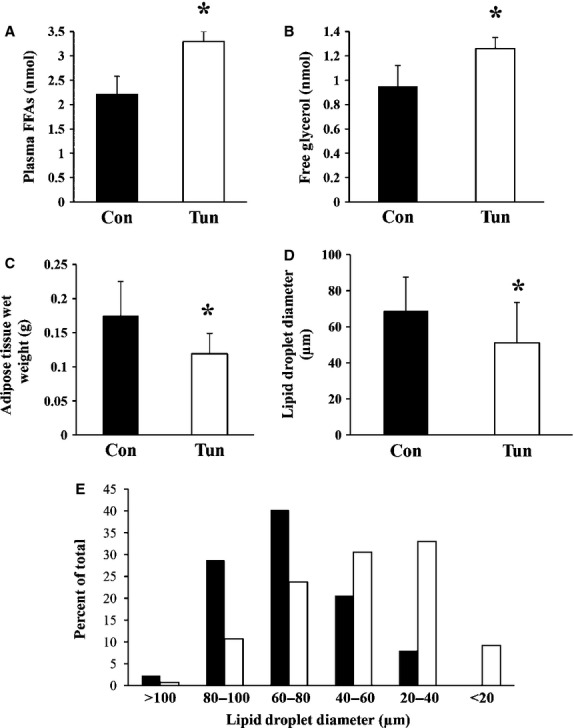
Male Balb/c mice were injected with either control buffer (Con) or tunicamycin (Tun) and sacrificed 24 hrs post injection. (**A**) Lipids were extracted from 35 μl of plasma and quantified. Values represent the mean ± SD, *n* = 5. (**B**) Plasma was deproteinized and free glycerol levels were determined. Values represent the mean ± SD, *n* = 3. (**C**) The epididymal fat pads were dissected above the epididymus and immediately weighed. Values represent the mean ± SD, *n* = 6 measurements. (**D**) The diameters of the lipid droplets within the adipocytes in the epididymal fat pads were determined. The values represent the mean ± SD of *n* = 87 (Con) and *n* = 131 (Tun) independent measurements obtained from three different mice per group. (**E**) Lipid droplet size distribution expressed as a percentage of the total number of droplets counted. ▪ Control, □ Tunicamycin. * indicates *P* < 0.05.

After we established that tunicamycin induced ER stress in adipose tissue, we next determined whether ER stress in adipose tissue was associated with increased lipolysis *in vivo*. Mice were injected with tunicamycin and sacrificed 24 hrs post injection. Tunicamycin increased circulating FFAs and glycerol levels by 49% and 30% respectively compared to control mice (Fig. 2A and B). Similar increases in FFAs were observed when mice were fasted for 4 hrs indicating that lipolysis occurred independent from nutritional intake (data not shown). Epididymal fat pads isolated from tunicamycn treated mice had significantly reduced wet weight compared to control mice (Fig. 2C) with no change in the wet/dry mass ratio (Control 1.25 ± 0.05, Tunicamycin 1.28 ± 0.06). The lipid droplets within the adipocytes of the epididymal fat pads were examined by electron microscopy. In control mice, the average diameter of the lipid droplets was ∼69 μm (median = 69.5 μm, range 20.4–102 μm). After 24 hrs following injection with tunicamycin the sizes of the lipid droplets decreased by ∼26% (mean = 51 μm, median = 51.8 μm, range 11.4–101 μm; Fig. 2D). Figure 2E shows the size distribution of lipid droplets measured in control and tunicamycin treated mice after 24 hrs and highlights the increased proportions of smaller lipid droplets following tunicamycin administration compared to control mice. A representative electron micrograph of lipid droplets within the epididymal fat pads and the associated size measurement is shown in Figure S4. The increase in lipolysis from adipose tissue stimulated by tunicamycin was not due to increased phosphorylation of HSL (Fig. S5). Examination of perilipin, a protein associated with the surface of lipid droplets that restricts lipolysis [29] was unchanged in the epididymal fat pads 24 hrs post injection with tunicamycin (Fig. S6).

### Tunicamycin induces fatty infiltration of liver, kidney, skeletal muscle and heart

**Fig. 3:**
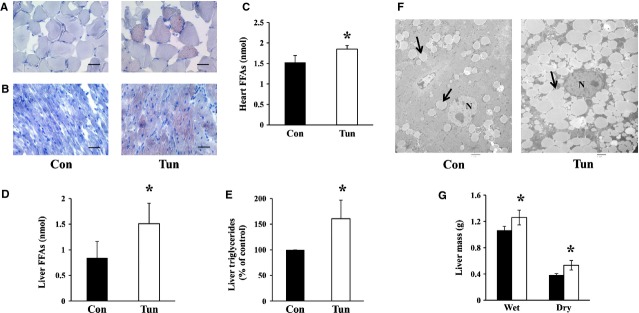
Male Balb/c mice were injected with either control buffer (Con) or tunicamycin (Tun) and sacrificed 24 hrs post injection. Oil Red O staining showing lipids (red) and nuclei (blue) in (**A**) gastrocnemius and (**B**) the heart, scale bar = 50 μm. (**C**) Whole hearts were dissected and lipids extracted and quantified as described. Values represent mean ± SD, *n* = 3. (**D**) Lipids were extracted from liver tissue and FFAs quantified. Values are mean ± SD, *n* = 6. (**E**) Liver triglyceride levels were quantified and expressed as a percentage of control. Values represent mean ± SD, *n* = 3. (**F**) Transmission electron micrographs of liver sections highlighting lipid droplets indicated by the arrows; scale bar = 2 μm, *N* nucleus. (**G**) Livers were dissected, weighed and placed in a dry 37°C incubator for 24 hrs. After drying, the liver was reweighed. Results represent the mean ± SD, *n* = 4–5. ▪ Control, □ Tunicamycin. * indicates *P* ≤ 0.05.

A hallmark of tunicamycin administration in mice is the rapid development of fatty livers with some lipid accumulation in the kidneys (Fig. S7). To determine whether there was lipid accumulation in other tissues after tunicamycin administration, we examined skeletal muscle and the heart. Following 24 hrs after injection with tunicamycin, lipids were accumulated in the gastrocnemius and heart indicated by Oil Red O staining (Fig. 3A and B). FFAs were extracted from whole hearts and found to be significantly higher in mice treated with tunicamycin (Fig. 3C). Interestingly, although FFAs were accumulated in the gastrocnemius and heart, ER stress was not detected in these tissues (Fig. S8). FFAs and triglycerides were also quantified in liver tissue. As expected, livers obtained from tunicamycin treated mice had a significantly increased FFA and triglyceride content compared to control mice (Fig. 3D and E). Liver tissue examined by transmission electron microscopy showed large and numerous lipid droplets within the cytoplasm of hepatocytes in tunicamycin treated mice (Fig. 3F). Tunicamycin treatment also led to significant increases in liver wet and dry mass (Fig. 3G).

**Fig. 4:**
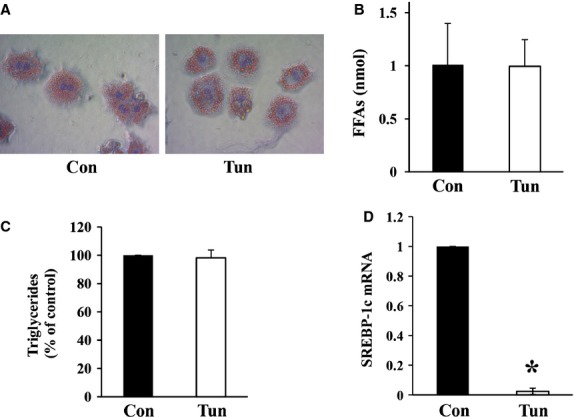
Primary mouse hepatocytes were isolated from Balb/c mice and incubated for 20–24 hrs in either media alone (Con) or media containing tunicamycin (5 μg/ml; Tun) and (**A**) stained with Oil Red O, Objective = 4×. Lipids were extracted from primary hepatocytes followed by quantification of (**B**) FFAs, values represent mean ± SD, *n* = 5 and (**C**) triglycerides, values represent mean ± SD expressed as a percentage of control, *n* = 3. (**D**) Quantification of SREBP-1c mRNA levels in livers 24 hrs following tunicamycin injection. Control livers were assigned a value of 1. SREBP-1c mRNA levels in tunicamycin treated livers are expressed relative to the control values. * indicates *P* ≤ 0.05.

To determine whether the accumulation of lipids within the liver was due to *de novo* lipogenesis induced by tunicamycin directly, primary hepatocytes were isolated and incubated with tunicamycin *in vitro* for 20–24 hrs at 37°C. Tunicamycin induced ER stress in primary cultured hepatocytes (Fig. S9) but did not induce significant lipid accumulation compared to control cells (Fig. 4). Examination of the mRNA levels of sterol regulatory element-binding protein 1c (SREBP-1c), a key transcription factor for the induction of lipogenesis in the liver, revealed significant reductions (∼40-fold) in livers from mice treated with tunicamycin (Fig. 4D). Taken together, these findings suggest that *de novo* lipogenesis likely does not account entirely for the accumulation of lipids within the liver following tunicamycin administration *in vivo*.

### ER stress in human adipose tissue following burn

Lastly, we attempted to corroborate our findings to a clinical scenario, burn injury. It is well recognized that burn patients suffer from profound hepatomegaly associated with increased lipolysis from adipose tissue [30,31]. We therefore examined whether ER stress is present in adipose tissue from burned patients.

**Fig. 5:**
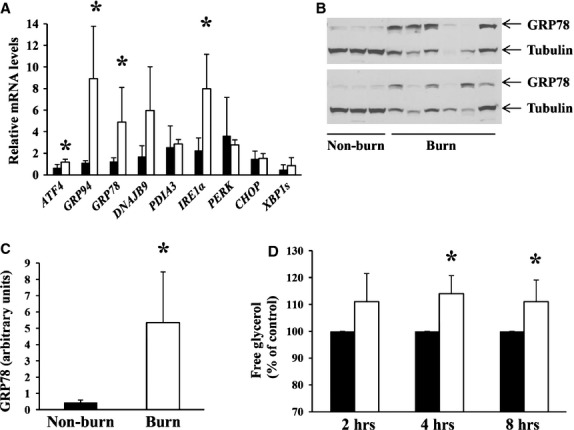
Subcutaneous white adipose tissue was obtained during surgery from burned patients (burn) or non-burned patients undergoing elective surgery (non-burn). (**A**) Total RNA was extracted from adipose tissue obtained from non-burned (*n* = 3–4) and burned patients (*n* = 5–8) and transcribed to cDNA. Real-time quantitative PCR was performed to evaluate the relative mRNA levels of the indicated genes that were normalized to *IDH1* mRNA. The value obtained from one non-burn sample was set to 1. The values obtained from all other samples were expressed relative to the non-burn sample. ▪ Non-burn, □ Burn. (**B**, *Top and Bottom Panels*) Adipose tissue was homogenized and equal amounts of total protein in the tissue homogenates were resolved by SDS-PAGE followed by immunoblotting using antibodies recognizing GRP78 and alpha/beta tubulin. (**C**) The intensities of the bands corresponding to GRP78 in (**B**) were quantified. (**D**) Human adipocytes (non-burn) were isolated and cultured either in the presence or absence of tunicamycin (5 μg/ml) at 37°C for the indicated times. The culture media was collected and the concentration of free glycerol determined. To account for the variability between patients in basal lipolysis rates, the amount of glycerol released from control cells from each patient and at each time-point was given a value of 100%. The amount of glycerol released from adipocytes in the presence of tunicamycin is expressed as a percentage of the value obtained from the control cells. Results represent the mean ± SD from *n* = 3 female patients ages 48 and 61 years, ▪ Control, □ Tunicamycin. * indicates *P* < 0.05.

Subcutaneous white adipose tissue was obtained from burned patients during surgery and from non-burned patients undergoing elective surgery. Adipose tissue from 26 burned patients (seven female, 19 male) was analysed. Total body surface area burned ranged from 4% to 78.5% and tissue was collected 1–12 days post burn. Adipose tissue obtained from non-burned patients (five female) served as controls. The ages of all patients ranged from 19 to 83 years. We first evaluated the presence of ER stress in adipose tissue from burned patients by examining a panel of ER stress markers by real-time quantitative PCR (Fig. 5A). Adipose tissue from burned patients showed significant up-regulation of *ATF4*, *GRP94*, *GRP78* and *IRE1*α compared to non-burned adipose tissue (Fig. 5A). To confirm the presence of ER stress at the protein level, we examined the up-regulation of GRP78. We previously determined that GRP78 is robustly up-regulated in human adipocytes under ER stress (Fig. S10). We therefore examined the expression of GRP78 in burned adipose tissue compared to non-burned. GRP78 was up-regulated in adipose tissue following burn in almost all samples compared to adipose tissue from non-burned patients (Fig. 5B and C). Figure 5B shows representative Western blots of GRP78 in adipose tissue obtained from 12 burned and three non-burned patients. Similar to mouse adipocytes, human adipocytes did not express detectable levels of PERK or CHOP (data not shown).

To determine whether ER stress would lead to augmented lipolysis in human adipocytes, adipocytes were isolated from non-burned patients (females aged 48 and 61 years) and incubated with tunicamycin *in vitro*. Tunicamycin rapidly induced lipolysis in cultured human adipocytes indicated by the accumulation of glycerol in the culture media after 2 hrs of incubation (Fig. 5D).

## Discussion

In this study, we show that the rapid development of fatty livers observed in mice following a single dose of tunicamycin could be due in part to increased lipolysis from adipose tissue. ER stress within adipose tissue is increasingly being recognized as a potential stimulus leading to augmented lipolysis [25,26]. A consequence of prolonged ER stress in adipose tissue where the increase in lipolysis is chronic would be the routing of lipids to other organs for storage [31]. The accumulation of lipids in the liver, kidney, heart and skeletal muscle following tunicamycin administration may reflect this possibility.

The mechanisms of how ER stress leads to augmented lipolysis are not entirely defined but a possible explanation could be the close relationship between the ER and lipid droplet [4,31]. The final steps of triglyceride synthesis take place at the ER and it has been has been suggested that the formation of lipid droplets occurs at the ER as well [32,33]. One model of lipid droplet formation proposes that neutral lipids accumulate within the ER membranes and after a critical lipid mass is reached the nascent lipid droplet pinches off together with the ER membranes [32,33]. Gross *et al*. [34] have recently shown that the transmembrane protein FIT2 located within the ER binds triglycerides and regulates lipid droplet size supporting the hypothesis that lipid droplets are derived from the ER. Once the lipid droplets are formed, the ER and lipid droplet are often located in close apposition, likely make membrane contacts and allow protein translocation between the two organelles [33]. White adipocytes contain a massive lipid droplet that occupies >90% of the adipocyte cell volume [35]. Given that the primary function of adipocytes is lipid storage and the close association between the ER and lipid droplet, it is possible that ER stress or disturbances in ER function would be reflected in functions related to the lipid droplet such as lipolysis/lipogenesis. Using cultured rat adipocytes, Deng *et al*. [25] showed that the ER stress inducer thapsigargin stimulated lipolysis *via* activation of the PKA signalling pathway and increased phosphorylation of HSL. Following tunicamycin administration, we did not observe consistent increases in HSL phosphorylation suggesting that increased activation of HSL may not be the primary mechanism of tunicamycin stimulated lipolysis. It could also be that the ER stress inducers thapsigargin and tunicamycin stimulate lipolysis by different mechanisms. Zhou *et al*. [26] showed that chronic ER stress in 3T3-L1 adipocytes led to decreases in perilipin levels. This is consistent with our observations obtained using primary human and mouse adipocytes incubated with tunicamycin *in vitro* (data not shown) although *in vivo*, chronic ER stress in mouse epididymal fat pads did not lead to decreases in perilipin levels. Perhaps the culture of adipocytes *in vitro* induces additional alterations that together with ER stress result in decreases in perilipin levels. Overall, given the complex interaction between the ER and lipid droplet it is difficult to identify a precise mechanism of how ER stress or ER dysfunction would lead to augmented lipolysis.

Within cells, tunicamycin inhibits *N*-linked glycosylation of proteins and lipids by binding to the enzyme *N*-acetylglucosamine-1-phosphate transferase and prevents the incorporation of *N*-acetylglucosamine into lipids and proteins [21,36]. It is not likely that interferences in protein/lipid glycosylation led to the stimulation of lipolysis in adipose tissue since numerous agents that induce ER stress such as thapsigargin, asymmetrical dimethylarginine and tunicamycin all enhanced lipolysis in adipose tissue but induce ER stress by different mechanisms [25,26]. It appears rather that ER stress signalling and the UPR augments lipolysis in adipose tissue. Interestingly, when cells take up glucose, a small fraction (2–3%) is metabolized in the hexosamine biosynthesis pathway for the generation of *N*-acetylglucosamine to provide precursors for glycosylation of proteins and lipids [37]. In adipose tissue, glucose deprivation inhibits glycosylation and up-regulates GRP78 [38] similar to the effects observed with tunicamycin. It seems logical to hypothesize that in adipose tissue nutrient (glucose) deprivation would induce ER stress and subsequently enhance lipolysis.

The observation that lipids accumulated mainly in the liver with much lesser amounts in the heart and muscle is likely because the liver is a central organ for the control of lipid metabolism. About 75% of blood flow to the liver is derived from the portal vein that carries blood from the gastrointestinal tract and the visceral fat [39]. This organization of blood flow ensures that the liver processes substances in the blood before delivery to the systemic circulation. This concept is supported by several publications demonstrating that white adipose tissue dysfunction leads to lipid accumulation in the liver and hepatic steatosis [40]. Consequently, the observation that lipid accumulation was highest in the liver upon elevated lipolysis stimulated by adipose tissue ER stress is not surprising. Skeletal muscle and the heart possess high oxidative capacities for FFAs that ensure energy production.

In burned patients, our observations and data obtained from a recent study [16] indicate that adipose tissue obtained post burn contains widespread changes in gene and protein expression compared to adipose tissue obtained from non-burned patients. For example, in the present study, the expression of tubulin was consistently decreased in burned adipose tissue compared to non-burned. The majority of proteins examined in our studies showed altered expression in burned adipose tissue suggesting that adipocyte function is significantly altered following burn. Similar alterations in protein expression were also noted when primary human and mouse adipocytes were incubated with tunicamycin for >20 hrs *in vitro* (data not shown). It is possible that chronic ER stress and/or ER dysfunction is the underlying cause.

Following a burn injury, a hypermetabolic response ensues that is characterized by hyperglycaemia, protein catabolism and increased lipolysis resulting in the increased availability of glucose, amino acids and lipids [30]. In the case of a burn injury, this hypermetabolic response is considered an adaptive response. Glucose and amino acids are primarily used for energy production. As a result of the profound hypermetabolism and most likely also because of the high levels of circulating catecholamines, lipolysis occurs [41]. Others and we believe that if the lipolysis response persists and remains uncontrolled, or if the oxidation of FFAs is insufficient, then lipids could accumulate in the liver [30,41]. The same scenario could be envisaged with tunicamycin administration where the lipolysis response in continual leading to a surplus of FFAs in the circulation and the accumulation of lipids in the liver as well as other organs. Elevations in circulating FFAs that occur as a result of enhanced lipolysis have shown to correlate with fatty infiltration of the liver [42]. In burned patients, hepatomegaly persists for 3 years and is associated with increased incidence of sepsis and infections [30]. Our study suggests that in addition to the catecholamines, ER stress in adipose tissue following burn may also play a role to augment lipolysis.

In summary, we found that ER stress in adipose tissue augments lipolysis *in vivo* and can subsequently lead to fatty infiltration of the liver and hepatomegaly. This prompts the idea of a novel treatment platform, where inhibiting lipolysis may improve outcome and organ function after injury. More specific and mechanistic studies are, however, needed to identify key components within the ER stress signalling pathways and the mechanisms that regulate lipolysis.

## Supporting information

**Figure S1.** Male Balb/c mice were injected with tunicamycin and sacrificed at the indicated times post injection.

**Figure S2.** Mice were injected with tunicamycin and after 24 hrs, the liver, kidney and epididymal fat pads were dissected and homogenized.

**Figure S3.** Epididymal fat pads and liver tissue were obtained from four mice (#1–4) and homogenized.

**Figure S4.** Representative electron micrograph of adipocytes within the epididymal fat pads.

**Figure S5.** Epididymal fat pads were dissected from male Balb/c mice at the indicated times after tunicamycin injection.

**Figure S6.** Epididymal fat pads were dissected from male Balb/c mice at the indicated times after tunicamycin injection.

**Figure S7.** Male Balb/c mice were injected intraperitoneally with either control buffer or tunicamycin.

**Figure S8.** Male Balb/c mice were injected with either control buffer or tunicamycin.

**Figure S9.** Tunicamycin induces ER stress in primary mouse hepatocytes.

**Figure S10.** Human adipocytes were isolated from a 48-year-old female patient undergoing elective surgery and incubated with tunicamycin (5 μg/ml) for 18 hrs at 37°C.

**Table S1.** Primer sequences.
