# Supplementary material for: Endoplasmic reticulum stress in adipose tissue augments lipolysis
Source: J Cell Mol Med. 2014 Nov 8;19(1):82–91. doi: 10.1111/jcmm.12384 (PMC4288352; doi:10.1111/jcmm.12384)
Supplement: Supplementary file 1 — Figure S1. Male Balb/c mice were injected with tunicamycin and sacrificed at the indicated times post injection. [file jcmm0019-0082-sd1.pdf]

Supplementary Figure 1

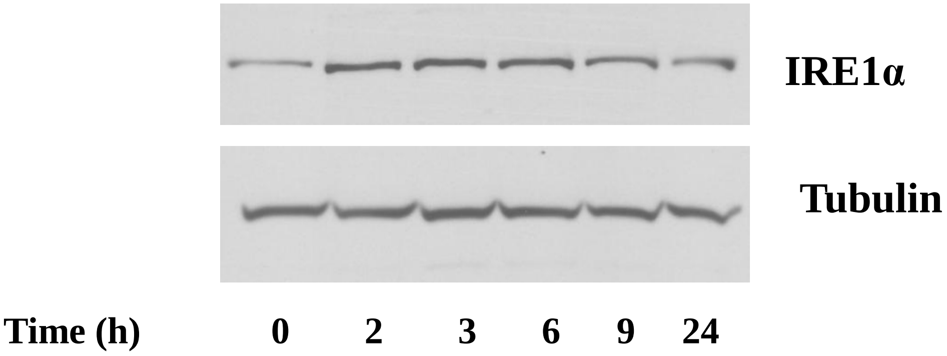

Supplementary Figure 1: Male Balb/c mice were injected with tunicamycin and sacrificed at the indicated times post injection. The epididymal fat pads were dissected and homogenized. Equal amounts of protein were resolved by SDS-PAGE and immunoblotted using antibodies recognizing IRE1α or alpha/beta tubulin. Proteins were visualized using enhanced chemiluminescence.
