# Supplementary material for: Endoplasmic reticulum stress in adipose tissue augments lipolysis
Source: J Cell Mol Med. 2014 Nov 8;19(1):82–91. doi: 10.1111/jcmm.12384 (PMC4288352; doi:10.1111/jcmm.12384)
Supplement: Supplementary file 2 — Figure S2. Mice were injected with tunicamycin and after 24 hrs, the liver, kidney and epididymal fat pads were dissected and homogenized. [file jcmm0019-0082-sd2.pdf]

Supplementary Figure 2

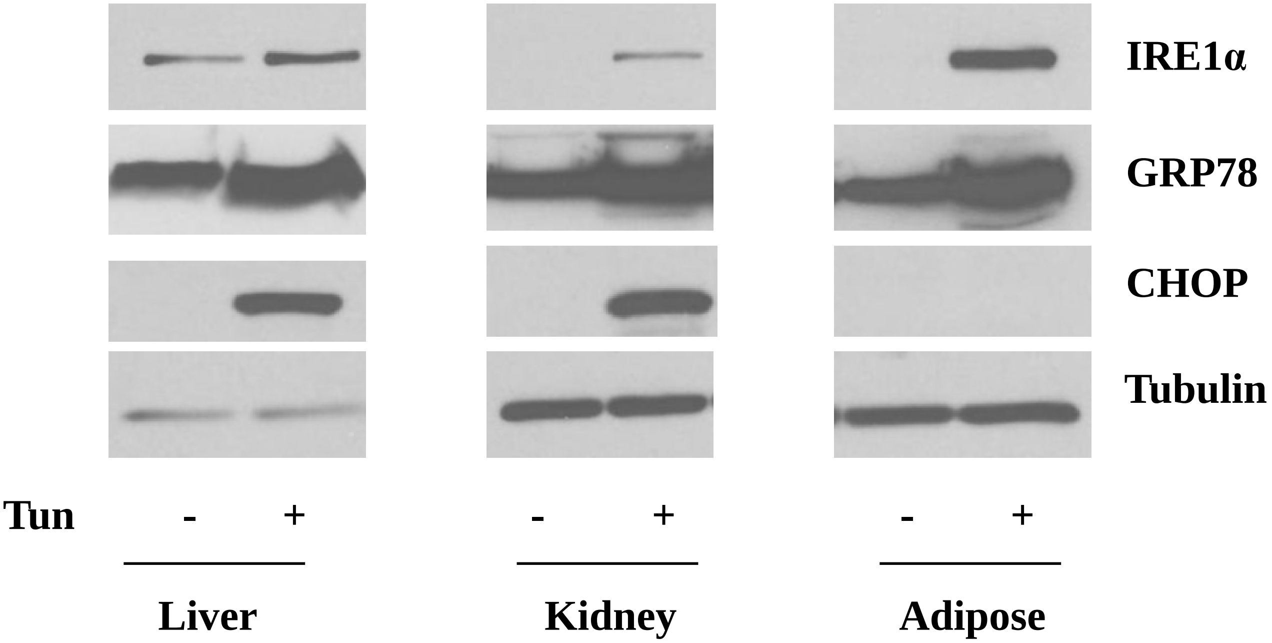

Supplementary Figure 2: Mice were injected with tunicamycin and after 24 h, the liver, kidney and epididymal fat pads were dissected and homogenized. Equal amounts of protein were resolved by SDS-PAGE and immunoblotted using antibodies recognizing IRE1α, GRP78, CHOP or alpha/beta tubulin. Proteins were visualized using enhanced chemiluminescence and imaged on the BioRad ChemiDoc MP Imaging System.
