# Supplementary material for: Endoplasmic reticulum stress in adipose tissue augments lipolysis
Source: J Cell Mol Med. 2014 Nov 8;19(1):82–91. doi: 10.1111/jcmm.12384 (PMC4288352; doi:10.1111/jcmm.12384)
Supplement: Supplementary file 3 — Figure S3. Epididymal fat pads and liver tissue were obtained from four mice (#1–4) and homogenized. [file jcmm0019-0082-sd3.pdf]

Supplementary Figure 3

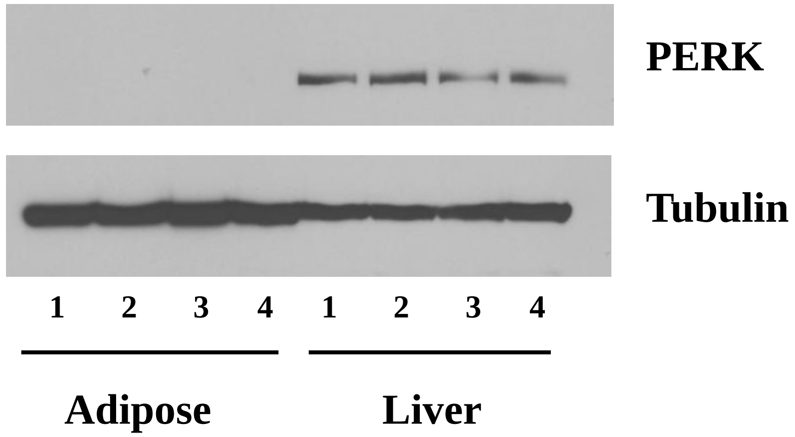

Supplementary Figure 3: Epididymal fat pads and liver tissue were obtained from four mice (#1-4) and homogenized. Equal amounts of protein were resolved by SDS-PAGE and immunoblotted using antibodies recognizing either PERK (Santa Cruz, H-300) or alpha/beta tubulin (Cell Signaling). Proteins were visualized using enhanced chemiluminescence.
