# Supplementary material for: Endoplasmic reticulum stress in adipose tissue augments lipolysis
Source: J Cell Mol Med. 2014 Nov 8;19(1):82–91. doi: 10.1111/jcmm.12384 (PMC4288352; doi:10.1111/jcmm.12384)
Supplement: Supplementary file 4 — Figure S4. Representative electron micrograph of adipocytes within the epididymal fat pads. [file jcmm0019-0082-sd4.pdf]

Supplementary Figure 4

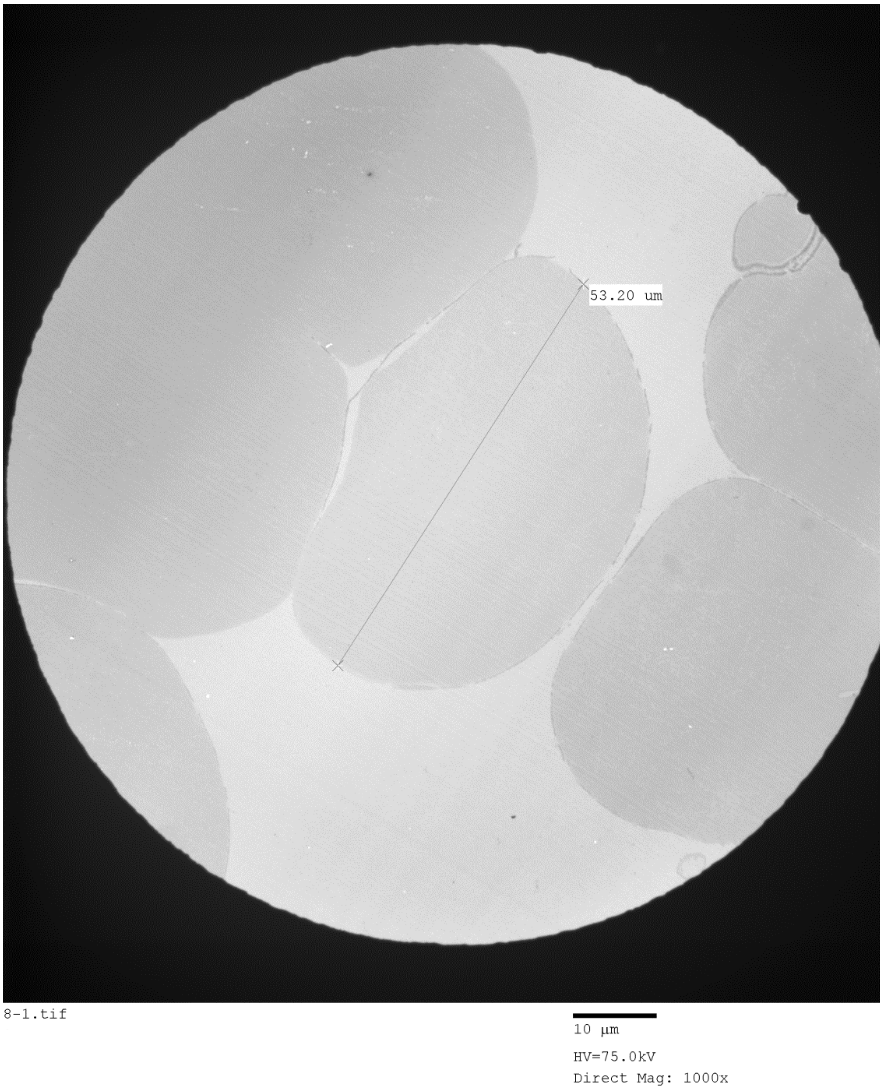

Supplementary Figure 4: Representative electron micrograph of adipocytes within the epididymal fat pads. The diameter of the lipid droplet is shown. Scale bar = 10µm.
