# Supplementary material for: Endoplasmic reticulum stress in adipose tissue augments lipolysis
Source: J Cell Mol Med. 2014 Nov 8;19(1):82–91. doi: 10.1111/jcmm.12384 (PMC4288352; doi:10.1111/jcmm.12384)
Supplement: Supplementary file 5 — Figure S5. Epididymal fat pads were dissected from male Balb/c mice at the indicated times after tunicamycin injection. [file jcmm0019-0082-sd5.pdf]

Supplementary Figure 5

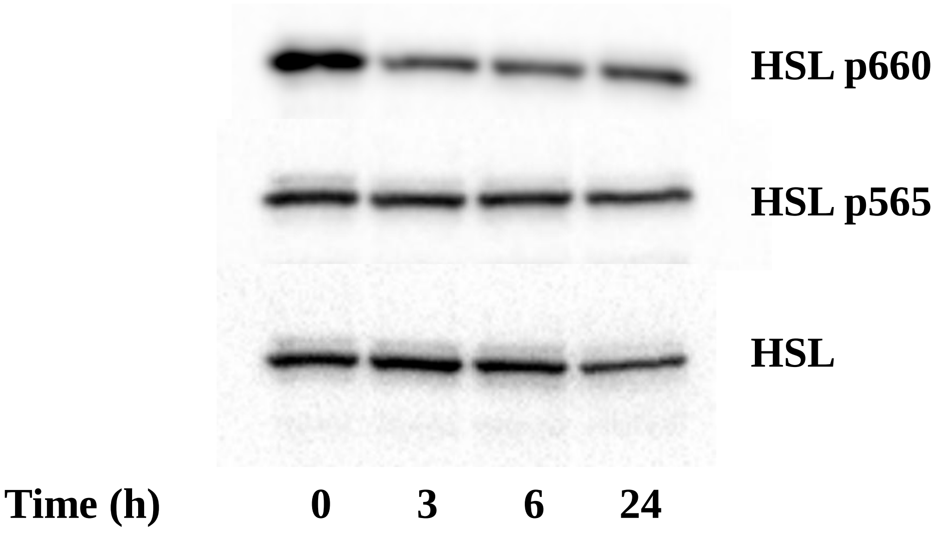

Supplementary Figure 5: Epididymal fat pads were dissected from male Balb/c mice at the indicated times after tunicamycin injection. The tissues were homogenized and equal amounts of protein resolved by SDS-PAGE followed by immunoblotting using antibodies recognizing the indicated proteins.
