# Supplementary material for: Endoplasmic reticulum stress in adipose tissue augments lipolysis
Source: J Cell Mol Med. 2014 Nov 8;19(1):82–91. doi: 10.1111/jcmm.12384 (PMC4288352; doi:10.1111/jcmm.12384)
Supplement: Supplementary file 6 — Figure S6. Epididymal fat pads were dissected from male Balb/c mice at the indicated times after tunicamycin injection. [file jcmm0019-0082-sd6.pdf]

Supplementary Figure 6

A

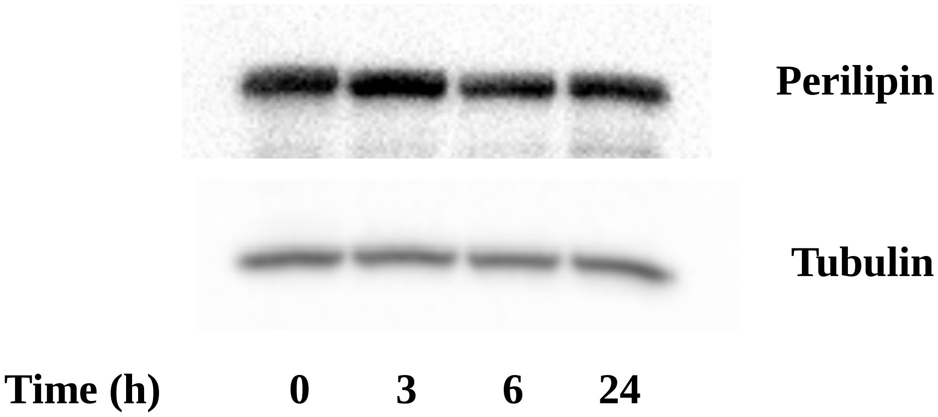

B

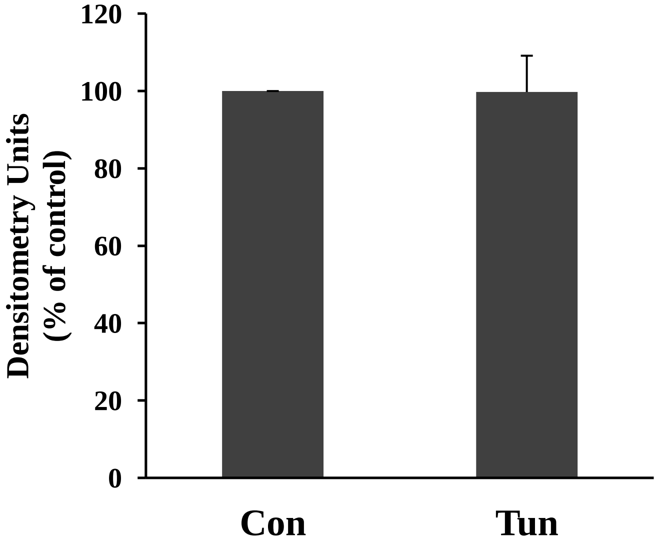

Supplementary Figure 6: (A) Epididymal fat pads were dissected from male Balb/c mice at the indicated times after tunicamycin injection. The tissues were lysed and equal amounts of protein resolved by SDS-PAGE followed by immunoblotting using antibodies recognizing the indicated proteins. (B) The intensities of the perilipin bands were quantified by densitometry. Graph represents mean±SD of values obtained at 24 h post injection, n=4.
