# Supplementary material for: Endoplasmic reticulum stress in adipose tissue augments lipolysis
Source: J Cell Mol Med. 2014 Nov 8;19(1):82–91. doi: 10.1111/jcmm.12384 (PMC4288352; doi:10.1111/jcmm.12384)
Supplement: Supplementary file 7 — Figure S7. Male Balb/c mice were injected intraperitoneally with either control buffer or tunicamycin. [file jcmm0019-0082-sd7.pdf]

Supplementary Figure 7

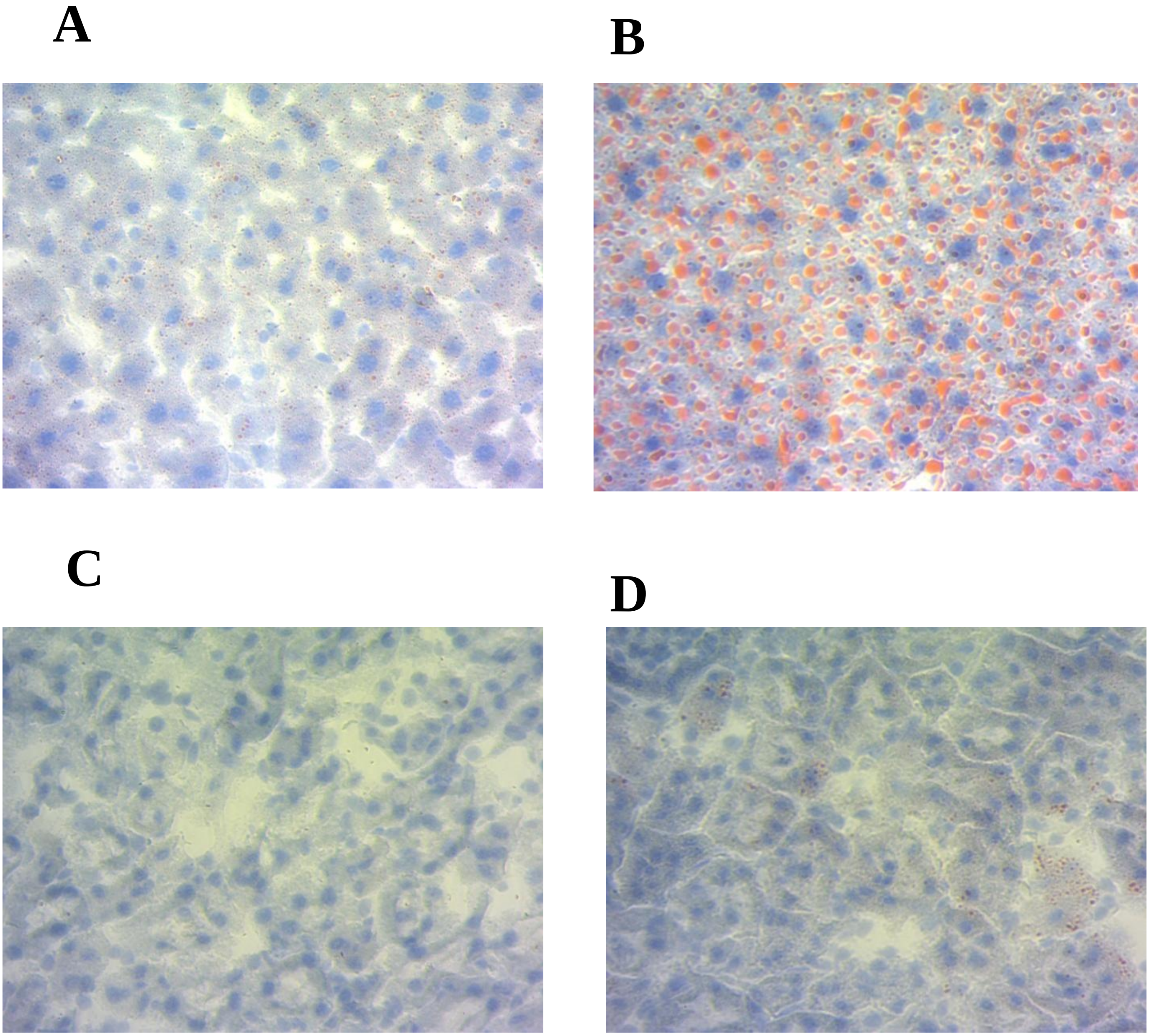

Supplementary Figure 7: Male Balb/c mice were injected intraperitoneally with either control buffer or tunicamycin. After 24 h, the liver and kidney were fixed and stained with Oil Red O and hematoxylin to visualize lipids (red) and nuclei (blue) respectively. (A) Control liver, (B) Tunicamycin treated liver, (C) Control kidney, (D) Tunicamycin treated kidney. Objective = 20x, n=5.
