# Supplementary material for: Endoplasmic reticulum stress in adipose tissue augments lipolysis
Source: J Cell Mol Med. 2014 Nov 8;19(1):82–91. doi: 10.1111/jcmm.12384 (PMC4288352; doi:10.1111/jcmm.12384)
Supplement: Supplementary file 8 — Figure S8. Male Balb/c mice were injected with either control buffer or tunicamycin. [file jcmm0019-0082-sd8.pdf]

Supplementary Figure 8

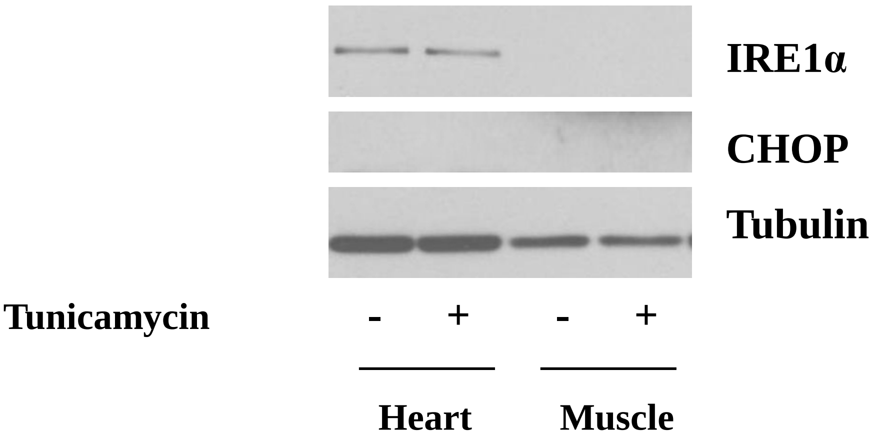

Supplementary Figure 8: Male Balb/c mice were injected with either control buffer or tunicamycin. After 24 h, the gastrocnemius and heart were dissected and homogenized. Equal amounts of protein were resolved by SDS-PAGE and immunoblotted using antibodies recognizing IRE1α, CHOP or alpha/beta tubulin. Proteins were visualized using enhanced chemiluminescence.
