# Supplementary material for: Endoplasmic reticulum stress in adipose tissue augments lipolysis
Source: J Cell Mol Med. 2014 Nov 8;19(1):82–91. doi: 10.1111/jcmm.12384 (PMC4288352; doi:10.1111/jcmm.12384)
Supplement: Supplementary file 9 — Figure S9. Tunicamycin induces ER stress in primary mouse hepatocytes. [file jcmm0019-0082-sd9.pdf]

Supplementary Figure 9

A

B

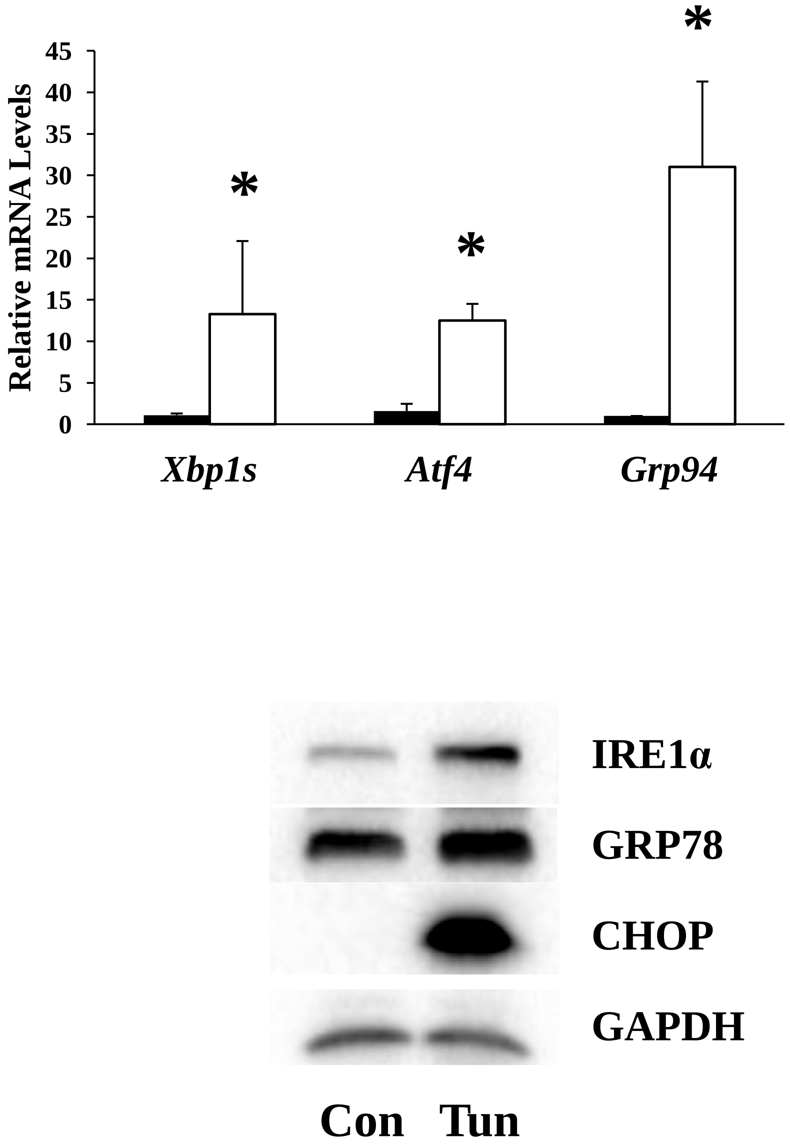

Supplementary Figure 9: Tunicamycin induces ER stress in primary mouse hepatocytes. Primary hepatocytes were isolated from male Balb/c mice and incubated for 20 h at 37°C in DMEM either in the absence (Con) or presence of tunicamycin (5µg/ml) (Tun). (A) Total RNA was extracted from cells, transcribed to cDNA followed by real-time PCR to quantify the changes in mRNA levels of the indicated genes. The value of each gene in control cells was assigned a value of 1. All other values are expressed relative to the control. ■ Control, □ Tunicamycin. * Indicates statistical significance $p < 0.05$. (B) Cells were lysed and equal amounts of protein were resolved by SDS-PAGE followed by Western blotting using antibodies recognizing the indicated proteins. Proteins were visualized using the BioRad ChemiDoc MP Imaging System.
