# Supplementary material for: Endoplasmic reticulum stress in adipose tissue augments lipolysis
Source: J Cell Mol Med. 2014 Nov 8;19(1):82–91. doi: 10.1111/jcmm.12384 (PMC4288352; doi:10.1111/jcmm.12384)
Supplement: Supplementary file 10 — Figure S10. Human adipocytes were isolated from a 48-year-old female patient undergoing elective surgery and incubated with tunicamycin (5 μg/ml) for 18 hrs at 37°C. [file jcmm0019-0082-sd10.pdf]

Supplementary Figure 10

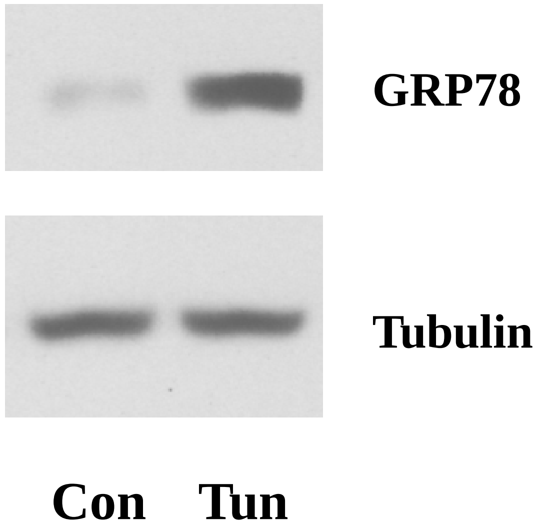

Supplementary Figure 10: Human adipocytes were isolated from a 48 year old female patient undergoing elective surgery and incubated with tunicamycin (5µg/ml) for 18 h at 37°C.
The cells were lysed and equal amounts of protein resolved by SDS-PAGE followed by immunoblotting using antibodies recognizing GRP78 or alpha/beta tubulin.
