# Supplementary material for: Endoplasmic reticulum stress in adipose tissue augments lipolysis
Source: J Cell Mol Med. 2014 Nov 8;19(1):82–91. doi: 10.1111/jcmm.12384 (PMC4288352; doi:10.1111/jcmm.12384)
Supplement: Supplementary file 11 — Table S1. Primer sequences. [file jcmm0019-0082-sd11.pdf]

Supplementary Table 1: Primer Sequences

**Gene**<br>**m=mouse, h=human**	**Primer Sequences**<br>Forward primer<br>Reverse primer
mIre1α	5'GACCGGCAGTTCCAGTACAT3'<br>5'TTGAGAGAATGCAGGTGTGC3'
mPerk	5'CCGATGTCAGTGACAACAGCTG3'<br>5'AAGACAACGCCAAAGCCACCAC3'
mAtf6	5'GTCCAAAGCGAAGAGCTGTCTG3'<br>5'AGAGATGCCTCCTCTGATTGGC3'
mGrp78	5'TGTCTTCTCAGCATCAAGCAAGG3'<br>5'CCAACACTTCCTGGACAGGCTT3'
mAtf4	5'TCCTGAACAGCGAAGTGTTG3'<br>5'ACCCATGAGGTTTCAAGTGC3'
mSrebp-1c	5'GCAGCCACCATCTAGCCTG3'<br>5'CAGCAGTGAGTCTGCCTTGAT3'
mGrp94	5'CTCACAGAGCCTGTGGATGA3'<br>5'TCTCTGTTGCTTCCCGACTT3'
mPdia3	5'GAGGCTTGCCCCTGAGTATG3'<br>5'GTTGGCAGTGCAATCCACC3'
mDnajb9	5'ATAAAAGCCCTGATGCTGAAGC3'<br>5'GCCATTGGTAAAAGCACTGTGT3'
mChop	5'CAGGGTCAAGAGTAGTGAAGGT3'<br>5'CTGGAAGCCTGGTATGAGGAT3'
mXbp1s	5'GAGTCCGCAGCAGGTG3'<br>5'GTGTCAGAGTCCATGGGA3'
18S rRNA	5' GTAACCCGTTGAACCCCATT3'<br>5' CCATCCAATCGGTAGTAGCG3'
hGRP94	5'CTGGGTCCAGCAGAAAAGAG3'<br>5'CTGGAACCTCTTCCCATCAA3'
hATF4	5'CCAACAACAGCAAGGAGGAT3'<br>5'GTGTCATCCAACGTGGTCAG3'
hGRP78	5'GCCGGCCAAGACAGCACAGA3'<br>5'GGGTCACAAGGCGCCACGAA3'
hXBP1s	5'GGGATGGATGCCCTGGTT3'<br>5'GCCTGCACCTGCTGCGGA3'
hDNAJB9	5'AGCGGCTACCTCCTGCCTG3'<br>5'GGAGCTGGCACGCACCCT3'
hCHOP	5'GCACCTCCCAGAGCCCTC3'<br>5'CCCGGGCTGGGGAATGAC3'
hPDIA3	5'TCCCAGGCCTACCCTGGTG3'<br>5'GCTCAGCTGCGTGGCAAG3'

hPERK	5'GTCCCAAGGCTTTGGAATCTGTC3'<br>5'CCTACCAAGACAGGAGTTCTGG3'
hATF6	5'CAGACAGTACCAACGCTTATGCC3'<br>5'GCAGAACTCCAGGTGCTTGAAG3'
hIRE1α	5'CCGAACGTGATCCGCTACTTCT3'<br>5'CGCAAAGTCCTTCTGCTCCACA3'
hIDH1	5'AAGGATGCTGCAGAAGCTATAA3'<br>5'CTTGAACTCCTCAACCCTCTTC3'
